# Geophysical monitoring and reactive transport modeling of ureolytically-driven calcium carbonate precipitation

**DOI:** 10.1186/1467-4866-12-7

**Published:** 2011-09-23

**Authors:** Yuxin Wu, Jonathan B Ajo-Franklin, Nicolas Spycher, Susan S Hubbard, Guoxiang Zhang, Kenneth H Williams, Joanna Taylor, Yoshiko Fujita, Robert Smith

**Affiliations:** 1 Earth Sciences Division, Lawrence Berkeley National Laboratory, 1 Cyclotron Road, Berkeley, CA 94720, USA; 2 Royal Dutch Shell International Exploration and Production Company, 200 N. Dairy Ashford Rd. Houston, TX, 77079, USA; 3 Center for Advanced Energy Studies, University of Idaho, 1776 Science Center Drive, Idaho Falls, ID 83402, USA; 4 Biological Systems Department, Idaho National Laboratory, P.O. Box 1625, Idaho Falls, ID 83415, USA

## Abstract

Ureolytically-driven calcium carbonate precipitation is the basis for a promising in-situ remediation method for sequestration of divalent radionuclide and trace metal ions. It has also been proposed for use in geotechnical engineering for soil strengthening applications. Monitoring the occurrence, spatial distribution, and temporal evolution of calcium carbonate precipitation in the subsurface is critical for evaluating the performance of this technology and for developing the predictive models needed for engineering application. In this study, we conducted laboratory column experiments using natural sediment and groundwater to evaluate the utility of geophysical (complex resistivity and seismic) sensing methods, dynamic synchrotron x-ray computed tomography (micro-CT), and reactive transport modeling for tracking ureolytically-driven calcium carbonate precipitation processes under site relevant conditions. Reactive transport modeling with TOUGHREACT successfully simulated the changes of the major chemical components during urea hydrolysis. Even at the relatively low level of urea hydrolysis observed in the experiments, the simulations predicted an enhanced calcium carbonate precipitation rate that was 3-4 times greater than the baseline level. Reactive transport modeling results, geophysical monitoring data and micro-CT imaging correlated well with reaction processes validated by geochemical data. In particular, increases in ionic strength of the pore fluid during urea hydrolysis predicted by geochemical modeling were successfully captured by electrical conductivity measurements and confirmed by geochemical data. The low level of urea hydrolysis and calcium carbonate precipitation suggested by the model and geochemical data was corroborated by minor changes in seismic P-wave velocity measurements and micro-CT imaging; the latter provided direct evidence of sparsely distributed calcium carbonate precipitation. Ion exchange processes promoted through NH_4_^+ ^production during urea hydrolysis were incorporated in the model and captured critical changes in the major metal species. The electrical phase increases were potentially due to ion exchange processes that modified charge structure at mineral/water interfaces. Our study revealed the potential of geophysical monitoring for geochemical changes during urea hydrolysis and the advantages of combining multiple approaches to understand complex biogeochemical processes in the subsurface.

## Background

**Figure 1:**
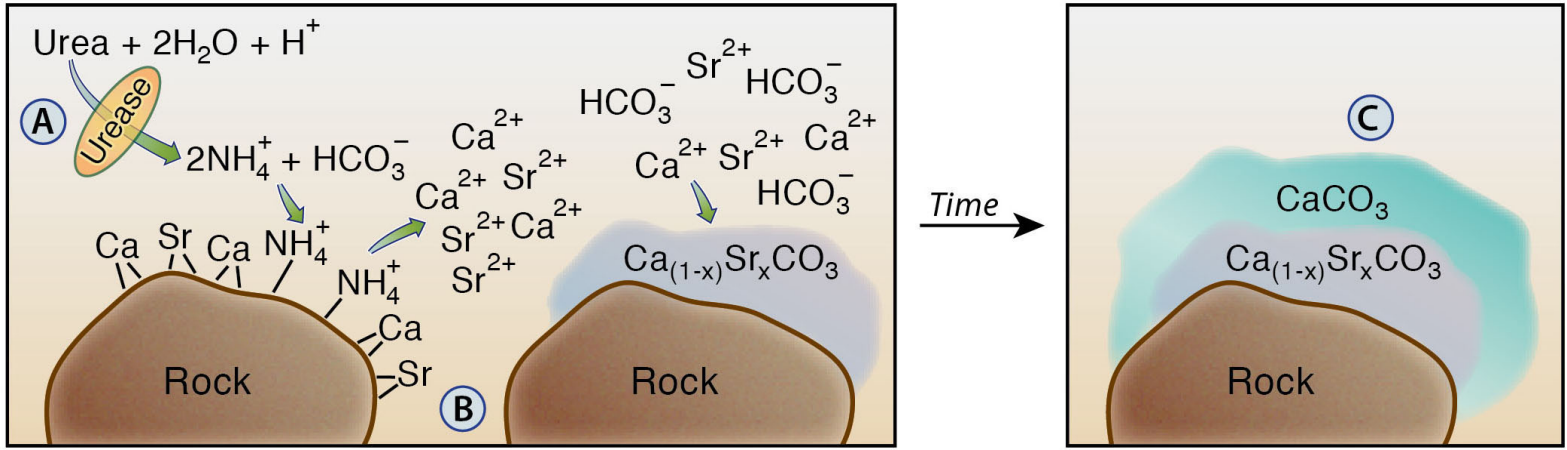
**Conceptual diagram of ureolytically-driven calcium carbonate precipitation approach for remediation of ^90^Sr contaminated geologic media**. A. Microbially catalyzed hydrolysis of urea. B. Cation exchange and calcium carbonate precipitation. C. Continued precipitation of calcium carbonate. Figure adapted from [8].

Remediation of subsurface radionuclides and trace metal contaminants is one of the U.S. Department of Energy's (DOE) greatest challenges for long-term stewardship [1]. Co-precipitation in calcium carbonate is one attractive *in-situ *remediation strategy for divalent radionuclide and trace metal ions, such as ^60^Co, ^137^Cs, and ^90^Sr [2-4]. The partitioning of these trace metals, e.g. ^90^Sr, into the calcium carbonate lattice is partly a function of precipitation rate [5-7]. One mechanism to promote calcium carbonate precipitation is through urea hydrolysis which increases groundwater pH and alkalinity, resulting in greater mineral saturation with respect to calcium carbonate. Urea hydrolysis is catalyzed by the urease enzyme, expressed by many microorganisms in the subsurface in order to harvest nitrogen. Figure 1 illustrates the conceptual model of the proposed reaction and contaminant sequestration processes [8].

The reactions involved in this process include:

1. Enzymatically catalyzed urea hydrolysis produces NH_4_^+ ^and HCO_3_^- ^and raises pH:

(1) $$H_{2}N\left(CO\right)NH_{2}+H^{+}+2H_{2}O\to 2NH_{4}^{+}+HCO_{3}^{-}$$

2. NH_4_^+ ^promotes desorption of cations M^2+^(e.g. Ca^2+ ^and Sr^2+^) from grain surfaces:

(2) $$Solid-\left(M^{2+}\right)+2NH_{4}^{+}\Leftrightarrow Solid-{\left(NH_{4}^{+}\right)}_{2}+M^{2+}$$

3. HCO_3_^- ^promotes precipitation of calcium carbonate and co-precipitation of ^90^Sr:

(3) $$x^{90}Sr^{2+}+\left(1-x\right)Ca^{2+}+2HCO_{3}^{-}\Leftrightarrow C{a_{\left(1-x\right)}}^{90}Sr_{x}CO_{3}+H_{2}O+CO_{2}$$

The net reaction leads to the production of carbonate minerals containing Sr:

(4) $$Solid-\left(x^{90}Sr^{2+}\right)+H_{2}N\left(CO\right)NH_{2}+\left(1-x\right)Ca^{2+}+2H_{2}O\to Ca_{\left(1-x\right)}Sr_{x}CO_{3}+Solid-{\left(NH_{4}^{+}\right)}_{2x}$$

Compared to direct injection of carbonate solution that could lead to clogging near the injection wells due to rapid precipitation of calcium carbonate, microbially facilitated calcium carbonate precipitation relies on the production of carbonate via urea hydrolysis, allowing urea to transport further into the subsurface before significant production of carbonate occurs. This could mitigate the risk of wellbore clogging and allow treatment of a larger area while using a single injection point. In addition, the NH_4_^+ ^produced during ureolysis can exchange with cations (including radionuclides such as ^90^Sr^2+^) previously sorbed to the subsurface materials (Eq. 2), thereby promoting metal sequestration in a more stable form (precipitation instead of sorption) (Eq. 3). This remediation approach is particularly attractive for environments where the groundwater is already saturated or oversaturated with respect to calcium carbonate, and thus the potential for releasing trapped metals through calcium carbonate dissolution over the long term is minimal. The arid western U.S. is an example of such an environment [9]. In addition to metal sequestration, enhanced calcium carbonate precipitation has many other potential applications, such as providing cementation for soil strengthening [10], and a long term sink for atmospheric carbon associated with CO_2 _storage/sequestration within geological media [11].

Although studies have demonstrated the potential usefulness of enhanced calcium carbonate precipitation for subsurface applications [10,12-15], one critical issue yet to be resolved is the monitoring of the calcium carbonate precipitation process in the subsurface to track its occurrence, spatial distribution, and temporal evolution. Such monitoring is necessary in order to evaluate its performance. In recent years, near surface geophysical methods have been used to provide high resolution imaging of subsurface properties in a minimally invasive manner, and these methods hold great promise for our ability to monitor subsurface perturbations [16,17]. Due to its sensitivity to mineralogy and pore fluid chemistry, electrical methods, e.g. complex conductivity, are among the most explored.

Complex conductivity (σ*) measures the frequency dependent electric conduction behavior of a porous medium. The measured σ*(ω) of a porous medium can be represented as,

(5) $$\sigma^{*}\left(\omega \right)=\sigma^{∕}\left(\omega \right)+i\sigma^{∕∕}\left(\omega \right).$$

where *ω *is the angular frequency, *σ' *is the measured real part of σ*(ω), being the conduction (energy loss) component; *σ'' *is the measured imaginary part of σ*(ω), being the polarization (energy storage) component and $i=\sqrt{-1}$. At low frequencies (< 1000 Hz), electrical charge transport in saturated porous media is determined by two processes: the electrolytic conductivity (*σ_el_*), representing conduction via interconnected fluid-filled pore space (a purely real term), and a complex interfacial conductivity (σ*_int_), representing conduction and polarization processes occurring near the grain/electrolyte interfaces. The *σ_el _*is dependent on the conductivity of the electrolyte (*σ_w_*) saturating the porous media [18] whereas σ*_int _represents an interfacial charge conduction and polarization behavior within the electrical double layer (EDL) at the grain/electrolyte interface dominated by a diffusive mechanism associated with ion migration to and from the grain surfaces [19-21]. The EDL is composed of (1) an inner fixed (Stern) layer with more or less structured charges adsorbed on the mineral surface through complexation and (2) an outer diffuse layer with more randomly distributed charges held weakly through electrostatic forces. Various factors could affect the interfacial charge conduction and polarization behaviors including mineralogical composition, specific surface area, particle size distribution as well as EDL properties, such as charge density, mobility and thickness. The EDL properties are influenced strongly by mineralogy and pore fluid properties, such as ionic speciation, concentrations, pH, etc. Other less explored factors that could change interfacial EDL properties include ion exchange behavior as well as the presence of organics [22].

When considering the polarization behavior at the grain/fluid interfaces of natural sediments, the inner Stern layer of the EDL structure is normally viewed as a relatively stable plane with primarily tangential movement of charges along the interface [21]. Charge movement normal to the interface is more difficult due to the higher potential barrier, the so-called zeta potential, that charges have to overcome in order to move away from or closer to the mineral surface [21]. Compared to the charges in the fixed layer, charge movement in the diffuse layer can occur more easily in either tangential or normal directions under external potential field due to the much weaker electrostatic forces. The polarization magnitude at the interface is primarily determined by the EDL properties (e.g. charge density, mobility). Alterations in pore fluid chemistry modify surface complexation structure, resulting in the changes of the EDL properties, thus polarization behavior [23]. This model has been successfully used to explain the polarization behavior of natural sediments. For example, under the condition of fixed solid matrix properties (mineralogy, surface area/particle size), previous research has shown that imaginary impedance (equivalent to *σ"*) changes in response to variations in ion concentrations in the pore fluid in Berea sandstone samples [24]. Other researches also demonstrated the sensitivity of electrical signals to changes in pore fluid chemistry and/or precipitation of new mineral phases due to amendment injection and mineral precipitation at both laboratory and field scales [17,25-29]. For example, Williams et al. [25] performed a laboratory biostimulation experiment where time-lapse complex conductivity, seismic, and various geochemical measurements were collected. They showed that changes in complex conductivity and seismic amplitude correlated with the onset and spatial distribution of microbially-mediated iron and zinc sulfide precipitation. Slater et al., [30], Ntarlagiannis et al., [27] and Personna et al [31] also showed the sensitivity of complex conductivity to metal sulfide precipitation/dissolution. These experiments demonstrated the potential of geophysical methods, particularly electrical measurement techniques, for the monitoring of mineral precipitation in porous media at the column scale. Recent studies have also illustrated the potential of geophysical methods to track remediation processes at the field-scale [16,17,28].

Particularly relevant to this study, Wu et al. [29] showed that abiotically induced calcium carbonate precipitation produced detectable geophysical signals during a laboratory column experiment. In that study, extensive and rapid calcium carbonate precipitation was induced through mixing of calcium and carbonate solutions in a glass bead packed column. Complex conductivity measurements collected daily during the experiment revealed a significant increase in the polarization magnitude, which coincided with the accumulation of calcium carbonate precipitates within the column and demonstrated clearly the sensitivity of the electrical signal to calcium carbonate precipitation. The polarization response observed was interpreted to be due to a large enhancement of the surface charge of the solid phase due to substantial calcium carbonate precipitation. However, because the experiment was conducted abiotically with a relatively high reaction rate and within an artificial medium, the sensitivity of geophysical methods to calcium carbonate precipitation at lower, field relevant rates and in the presence of field derived sediments warrants further evaluation.

In this study, we conducted laboratory flow-through column experiments to explore sensitivity of geophysical methods to ureolytically-driven calcium carbonate precipitation under field relevant conditions with natural sediments, groundwater and microbial consortium. Primary mechanisms by which microbially enhanced calcium carbonate precipitation through urea hydrolysis could change both the conduction and polarization components of the complex conductivity through (1) changing the ionic strength/speciation and pH of the pore water (and thus the surface charge properties of the solid), (2) increasing the total surface area of the solid matrix through the precipitation of calcium carbonate, and (3) changing the hydraulic conductivity/permeability as a result of changes in the pore geometry (if significant calcium carbonate precipitation occurs). In addition to contributions to the increases in ionic strength, NH_4_^+ ^produced during urea hydrolysis (Eq. 1) could engage in ion exchange with the sediments (Eq. 2), thereby providing additional polarization effects through altering surface charge structure by (1) changing surface charge density through non-stoichiometric charge exchange and (2) altering complexation structure between surface sites and absorbed species.

In addition to electrical signals, seismic wave propagation velocities have proven to be moderately sensitive to bioprecipitate formation with previous studies documenting changes in P-wave attenuation during FeS precipitation [25,32] and significant increases in S-wave velocity during CaCO_3 _precipitation induced by microbial urea hydrolysis have also been observed [13]. Generally, changes in seismic velocities are due to stiffening of the granular framework, a process that should be captured by existing contact cement theories (CCT) [33]. Replacement of pore fluids by a solid mineral phase at weak grain contacts results in an increase in both bulk and shear modulus during the precipitation process. Secondary changes in seismic properties related to poroelastic phenomenon, including so-called double porosity effects [34], may also be present in this class of systems due to heterogeneous precipitation or micro-porosity within the precipitate phase.

In addition to geophysical monitoring, 1D reactive transport modeling using TOUGHREACT ([35] and references therein) was carried out to simulate reactions occurring during the experiment. Geochemical processes considered in the simulations included ureolysis together with calcium carbonate precipitation, ion exchange, and nitrification. The effect of dissolution and precipitation of minerals other than calcium carbonate was assumed to be negligible during the relatively short time frame of the experiment.

Dynamic synchrotron x-ray micro-CT measurements were conducted to characterize precipitate distribution to inform analysis of observed geophysical signatures. X-ray micro-CT is a non-destructive 3D imaging methodology with micron scale resolution which has been applied to the characterization of porous solids since the mid-1990s [36,37]. By dynamic, we refer to multiple CT images captured over a period of time to allow process monitoring [38]; in this case, the process is the formation of CaCO_3 _precipitates. The micro-CT technique is directly analogous to medical CT scanning with the exception of the greatly enhanced resolution, smaller imaging target, and a different x-ray source. In the case of precipitate generation within a porous medium, a solid mineral phase is replacing the pore fluid, a change easy to observe using micro-CT methods. In this particular study, we use dynamic micro-CT data qualitatively to characterize the spatial pattern of precipitation on the pore scale. The key questions that we address include the spatial distribution, morphology, and amount of precipitates formed.

We anticipated coupling between and uncertainty associated with several processes, including: the magnitude and spatial distribution of calcium carbonate precipitates; the clogging of pore throats and concomitant change in flow properties associated with precipitation [39]; and temporal variation of geophysical signatures associated with different phases of the reaction processes. The combination of geophysical datasets, reactive transport modeling, micro-CT imaging and jointly interpreted monitoring and modeling datasets helped reducing ambiguities associated with these processes. Specifically, dynamic synchrotron x-ray micro-CT measurements provided direct information about the magnitude and nature of calcium carbonate precipitation at the pore scale including its geometric arrangements within pores and spatial distribution throughout the column. Complex conductivity and seismic P-wave velocity measurements were used to gain understanding about effective changes in geochemical and geomechanical conditions, respectively, averaged over the length of the column. Reactive transport modeling helped to understand the rates associated with the reaction network described in Equations 1-4 and to predict the volume of produced calcium carbonate. Together, the methods were used to gain an improved predictive understanding of ureolytically-driven calcium carbonate precipitation in natural media and particularly, of the potential of geophysical methods for monitoring the relevant biogeochemical changes over space and time.

## Methods

### Column setup

Two columns were fabricated for the experiment from polycarbonate plastic: a large multi-port column for electrical and seismic measurements and a second smaller column for synchrotron micro-CT imaging. The larger column has a length of 20 cm and 7.6 cm inner diameter (I.D.) while the micro-column has a length of 12.7 mm and an I.D. of 6.35 mm.

Alluvial sediments used in the experiment were collected from a background site north of Idaho Nuclear Technology and Engineering Center (INTEC) of the Idaho National Laboratory (INL). ^90^Sr contamination was found in the subsurface below the INTEC facility. Groundwater used in the experiment was from the Vadose Zone Research Park (VZRP) at the INL. The VZRP subsurface has geological and hydrological properties similar to those at the nearby INTEC. The VZRP groundwater is not radiologically contaminated but has naturally occurring nonradioactive Strontium that can be used to study the co-precipitation behavior during ureolytically-driven calcium carbonate precipitation.

For the large electrical/seismic column, the sediments were used after large pebbles (> 1 cm) had been removed. This sediment had been archived and dried for over ten years. Significant clay content ranging from 8% to 28% has been measured for core retrieved from the site, depending on locations and depth; illite and smectite are among the major clay minerals identified [40,41]. The dry material was saturated in VZRP groundwater (fluid conductivity, *σ_w _*~ 0.062 S/m) overnight and then wet packed into the column. A 6 cm section of coarse silica sand (particle size ~1 mm) was packed at the bottom of the column to facilitate homogeneous fluid flow into the sediment. In an effort to maximize homogeneity, the sediments were packed ~ 1 cm at a time and tapped from outside to allow settlement and compaction [42]. However, seismic data revealed a certain level of heterogeneity along the column length (discussed below). The smaller micro-CT column was packed using the same protocol except that the sediment was sieved to include only particles less than 1 mm in size; this modification was chosen due to the small I.D. of the column (6.35 mm). An initial hydraulic conductivity (*K*) measurement was carried out on the electrical/seismic column using the constant head method and a *K *value at 1.2E-5 m/s was measured. This value is within the range of values previously measured on core samples retrieved from boreholes drilled at the INL (varying from 1E-5 to 1E-9 m/s depending on depth and location) [40].

The experiment was carried out in three phases: Phase I: groundwater was pumped through the columns to achieve an initial steady state as defined by stability of effluent conductivity; Phase II: groundwater amended with molasses (5 mg/L) was injected into the columns for ~3 days to stimulate microbial growth and activity [15]; and Phase III: groundwater amended with urea (10 mM) was delivered into the columns to induce urea hydrolysis and calcium carbonate precipitation. The molasses concentration was purposefully kept low to stimulate microbial activities at an appropriate level, (i.e. to prevent pore clogging by excessive precipitation or the transition into a sulfate reduction phase).

**Figure 2:**
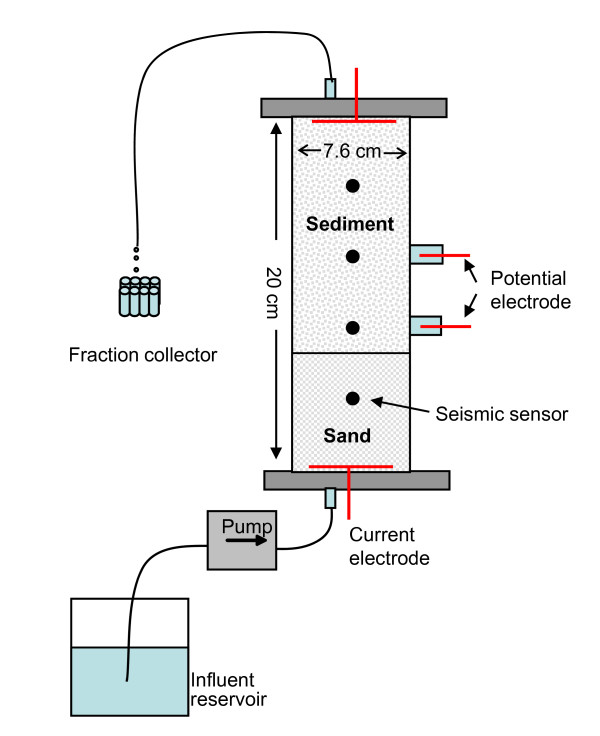
**Schematics of the flow through column design**. Black dots represent locations of the seismic transducers and electrodes are indicated in red.

For the large column experiments, complex conductivity measurements were acquired using a four electrode configuration, with one electrode installed inside each end cap for current injection and two installed in the middle of the columns for measurements (Figure 2). Ag/AgCl electrodes were used for both current injection and potential measurements. Spiral current electrodes inside the end caps were in direct contact with column material while potential electrodes were electrolytically coupled with the column through small plastic tubes installed on the column wall and filled with the groundwater saturating the column. The electrodes recovered after the experiment had no visible alterations (e.g., mineral precipitation or dissolution of AgCl coating). For the measurements of the seismic signals, four pairs of P-wave seismic transducers (1 MHz) were installed along the column length in an orientation 90 degrees from the Ag/AgCl potential electrodes. Each transducer was coupled to the sediment through a 0.5 cm aluminum buffer rod which directly contacted the material within the column.

A constant flow rate of 0.21 ml/min (~0.7 pore volumes per day with a measured porosity of 45%) was used throughout the experiment for the large column, which resulted in a residence time of ~32 hours. A fraction collector (Foxy 200, ISCO) was used to collect effluent samples on regular basis from the large column. Hydraulic conductivity measurements were conducted at the end of the experiment to determine the change in hydraulic conductivity from calcium carbonate precipitation. The injection rate for the micro-column was selected to match the fluid residence time (0.7 pore volumes/day) in the larger column and the same influent supply was used for both columns.

### Geophysical methods

Electrical measurements were collected with a National Instruments (NI) dynamic signal analyzer (DSA, NI 4461) using electrodes placed along the length of the large column (described above). A preamplifier was used to boost the input impedance to 10^9 ^Ohm to avoid current leakage into the measurement circuitry. Water column calibration and repeatability tests indicate that errors were less than 0.3 mrads for the phase and 0.5% for conductivity when low frequency was used (< 500 Hz). Each measurement is composed of a phase shift (*Φ*) and a magnitude (|*σ|*) component recorded relative to a precision reference resistor for forty frequencies spaced at equal logarithmic intervals from 0.1 to 1000 Hz. The real and imaginary parts of the complex conductivity represent the magnitude of the conduction and polarization of the sample respectively, and were determined from the following equations:

(6) $$\sigma^{\prime }=\left|\sigma \right|\times \cos\phi$$

(7) $$\sigma^{\prime \prime }=\left|\sigma \right|\times \sin\phi$$

In this study, we also measured P-wave velocity and amplitude using the ultrasonic transmission method at ~1 MHz. At several points along the length of the column, piezoelectric compression transducers mounted opposite each other allowed a through-column measurement of P-wave traveltime at regular time intervals during the experiment. The transducers were excited using a broadband ultrasonic pulser (Panametrics Model 5075) and the transmitted signal was recorded via a digital oscilloscope (Tektronix Model TDS210). The resulting transmitted waveforms were picked using a customized Graphic User Interface (GUI) and then corrected for zero-time and column shell delays. The times were then converted to P-wave velocities using the known transmission distance.

### Geochemical monitoring

Geochemical analysis of effluent samples from the large column was conducted on regular basis. Electrolyte conductivity (*σ_w_*) and pH were measured near real time with a pH/conductivity meter (Orion 5 star, Thermo Scientific). Effluent dissolved oxygen (DO) was measured with a YSI Model 5000 DO meter and was used as an proxy indicator of microbial activity during molasses injection and was measured periodically to ensure sustained microbial activity afterwards during urea injection. Major anions (Cl^-^, SO_4_^2- ^and NO_3_^-^) were measured with ion chromatography (IC, Dionex) and major cations were measured with Inductively Coupled Plasma Mass Spectrometry (ICP-MS, Perkin Elmer). Alkalinity was measured using an acid titrimetric method (Mettler Toledo DL 50 Graphix titrator). Effluent ammonia and urea concentration were also quantified using IC analysis. Due to the automated sampling procedure with the fraction collector and the equilibrium of the sample fluid with atmospheric pCO_2_, partial NH_4_^+ ^oxidation (production of NO_3_^-^) in the effluent and premature calcium carbonate precipitation in the influent container were observed and both issues were accounted for in the modeling studies.

### Micro-CT imaging

A 3^rd ^generation synchrotron, the Advanced Light Source (ALS) at Lawrence Berkeley National Laboratory was used as the x-ray source for micro-CT imaging. Synchrotron x-ray sources are attractive for micro-CT studies because of their extremely high flux and parallel beam; the former feature allows for use of a monochromator avoiding beam hardening artifacts. All imaging for our experimental effort was conducted at ALS beamline 8.3.2, which is positioned on a super-bend magnet and provides access to x-ray energies between 8 and 41 keV. The resulting x-ray attenuation image stack is sensitive to changes in localized density or atomic number, thus allowing discrimination between solid, fluid, and gas phases as well as some differences in solid mineral phases.

The micro-CT acquisition protocol includes careful mounting of the experimental subject on a rotating stage and acquisition of a sequence of x-ray projections over a range of 180 degrees. X-rays passing through the sample are converted to visible light at a scintillator, and the resulting light passes through a traditional microscope optics chain and is recorded using a high-resolution CCD (Cooke PCO4000). This sequence of radiographs is then inverted using a filtered back-projection algorithm (Octopus, InCT Inc.) to generate a 3D volume of x-ray absorptivity. A 2× optical objective (Mitutoyo) was used during scan yielding final 3D reconstructed volumes with cubic voxels of ~4.47 microns. The entire length of the column was scanned for both the baseline and repeat imaging runs.

### Destructive sampling

The columns were destructively sampled after the experiments for multiple characterization purposes. The large geophysical column was sub-cored with polycarbonate tubing for micro-CT imaging and comparison with the miniature column to characterize geometry and spatial distribution of the precipitates. Both pore fluids and fine sediment samples from the column were characterized for cell density after staining with acridine orange and direct counting using a Zeiss Axioskop fluorescence microscope. In addition, cell adherence to sediment particles was determined through washing with CaCl_2 _and Tween 80/pyrophosphate solutions to neutralize cell surface charge and promote cell detachment [43] and cells in supernatant wash fluids were counted after staining. Recent research found that ureolytic activity on a per gram or per ml basis was much higher on the sediments than in the fluid [8]. Cell detachment analysis provides information of relative abundance of cells attached to sediments comparing to those in the fluid, and can be used to evaluate relative ureolytic activity of these cells.

### Reactive transport modeling

#### Model Setup and Approach

The numerical code TOUGHREACT ([35] and references therein) was used to simulate reactions occurring during the flow through column experiment. A 15-day period was simulated, starting at the time of urea-amended groundwater injection (Phase III). The initial sediment pore water was taken as the measured water composition at the column outlet after the initial equilibration period (Phase I), but prior to urea injection (Table 1). However, the elevated measured pH (~8.6) at the column outlet at the end of Phase I indicated partial equilibration with atmospheric CO_2 _upon sampling and supersaturation of the solution with respect to calcium carbonate. To better represent conditions within the column, the initial total carbonate concentration and pH within the column were set to reflect equilibrium with calcium carbonate, which yielded pH and total dissolved carbonate values (Table 1) more in line with pH values determined for VZRP groundwater.

**Table 1:** Water compositions used in simulations

Analyte	Units	Influent composition before Phase III	Influent composition during phase III
Na	mM	3.05	3.03
Mg	mM	0.466	0.487
Al	mM	0.28 × 10^-3^	0.15 × 10^-3^
Si	mM	0.334	0.343
K	mM	0.087	0.066
Ca	mM	1.05	1.00
Sr	mM	2.82 × 10^-3^	2.83 × 10^-3^
Cl	mM	2.63	2.63
NO_3_	mM	44.5 × 10^-3^	18.1 × 10^-3^
SO_4_	mM	0.213	0.206
pH		7.62^(a)^	8.23
ΣCO_3_^(a)^	mM	3.23 ^(a)^	4.21 ^(a)^
*p*_CO2_	bar	10^-2.37 (a)^	10^-3.01 (a)^
Urea	mM	nil (10^-10^)	10

^a ^Calculated (see text)

The pH measured in the influent reservoir was higher (~ 8.2) than that observed in the VZRP (~7.6) where the groundwater used in the experiment was acquired, also indicating some re-equilibration with atmospheric CO_2 _in the reservoir. In this case, the composition of the injected water (column inlet) was specified using the actual measured pH and species concentrations in the influent reservoir, but correcting the total dissolved carbonate concentration to yield ion charge balance (Table 1).

Geochemical processes considered in the simulations included ureolysis together with calcium carbonate precipitation, ion exchange, and nitrification, as further discussed below. The effect of dissolution and precipitation of minerals other than calcium carbonate (such as silicates and aluminosilicates) was assumed to be negligible during the relatively short time frame of the experiment on the basis that the reaction rates of these minerals are much slower than calcite reaction rates [44]. Calcium carbonate was allowed to precipitate in the sand pack, but this part of the column was otherwise assumed unreactive, with essentially no ion exchange capacity.

The model domain was set as a 1D cylindrical column with dimensions and hydraulic properties described above. A fixed influx rate (0.21 mL/min) was applied at the modeled column inlet. The column was discretized into 205 grid blocks at regularly spaced intervals of 1 mm. A sequential-iterative (transport/reaction) method was implemented to reproduce accurate concentration time profiles, using a maximum time step of 416 s (~0.8 × Courant). Tests using finer time and space intervals indicated that this set of parameters provided a reasonable compromise between simulation speed and accuracy. To allow for partial re-equilibration of effluent water with atmospheric CO_2_, the modeled column outlet was set at a constant *p*CO_2 _of 10^-3.3 ^bar, a value estimated (by trial and error) to yield best results; CO_2 _was not allowed to diffuse back into the column. Automatic calibration of other model parameters was performed using PEST [45] on a Unix cluster, including the total enzyme concentration driving the hydrolysis of urea (including EH species in Eq. 8, 9 and 10 shown below), a similar concentration term driving the rate of nitrification (*C_bio_*/*Y_bio _*in Eq. 13 shown below), the calcium carbonate precipitation rate constant, and the ion exchange capacity of the sediments.

#### Ureolysis kinetic rate law

Ureolysis was modeled as an enzymatic reaction, with a rate law taking into account the effect of pH on enzyme (urease) activity as well as product inhibition by NH_4_^+ ^[46]:

(8) $$R=k\frac{\left[\text{EH}\right]\left[\text{Urea}\right]}{\left(K_{\text{M}}+\left[\text{Urea}\right]\right)\left(1+\frac{\left[{\text{NH}}_{4}^{+}\right]}{K_{p}}\right)\left(1+\frac{10^{-\text{pH}}}{K_{1}}+\frac{K_{2}}{10^{-\text{pH}}}\right)}$$

In this equation, brackets indicate concentrations, EH represents the enzyme driving the hydrolysis of urea, *K_M _*and *K_P _*are the half-saturation and inhibition constants, respectively, and *k *is a rate constant. *K_1 _*and *K_2 _*are dissociation constants representing the enzyme protonation and de-protonation reactions, respectively. This rate law was implemented into TOUGHREACT, as described previously [47], using a standard Michelis-Menten rate law with an inhibition term:

(9) $$R=k\left[\text{EH}\right]\frac{\left[\text{Urea}\right]}{K_{\text{M}}+\left[\text{Urea}\right]}\frac{K_{\text{P}}}{K_{\text{P}}+\left[{\text{NH}}_{4}^{+}\right]}$$

A primary species, EH, was added to the input thermodynamic database to represent the enzyme, together with two additional derived species, EH_2_^+ ^and E^-^, expressing the protonated and deprotonated enzyme species:

(10) $$\begin{array}{cc} {\text{EH}}_{2}^{+}↔\text{EH}+{\text{H}}^{+} & \log{\left(K_{1}\right)}_{25^{\circ }\text{C}}=-6.121 \end{array}$$

(11) $$\begin{array}{cc} {\text{E}}^{-}+{\text{H}}^{+}↔\text{EH} & \log{\left(K_{2}\right)}_{25^{\circ }\text{C}}=7.896 \end{array}$$

It can be shown that this approach is a mathematical equivalent to using Equation 8. Values of *K_1 _*and *K_2 _*were taken from Fidaleo and Lavecchia [46]. The total concentration [EH] + [EH_2_^+^] + [E^-^] was assumed constant and calibrated (10^-10 ^mol_urease_/L). Other rate law parameters were taken from Fidaleo and Lavecchia [46] as follows (at 25°C, with *k *re-expressed using molar concentrations): *K_P _*= 1.22 × 10^-2 ^mol/L, *K_M _*= 3.21 × 10^-3 ^mol/L, and *k *= 146.4 mol _urea _mol_urease_^-1 ^s^-1^.

#### Calcium carbonate precipitation

In the model, calcium carbonate (CaCO_3_) was allowed to form as an ideal solid solution with strontianite (SrCO_3_) (i.e., assuming equality of activity and mole fraction). The solid solution was set to precipitate under kinetic constraint using a rate constant calibrated from the experimental data. A standard rate law derived from Transition State Theory (TST) was implemented [48], in which the rate constant is multiplied by (1-*Q/K*), with *Q *and *K *representing the ion activity product and equilibrium constant, respectively, of the calcium carbonate/strontianite solid-solution precipitation reaction. Nucleation was not specifically modeled, and the rate constant was calibrated as a bulk average rate (4.2 × 10^-10 ^mol/s) incorporating surface area effects. Although this approach is quite approximate, it yielded satisfactory results. More elaborate, mechanistic precipitation models were deemed unwarranted given that the uncertainty of the calcium carbonate precipitation rate is large and partly carried through the calibration of other parameters.

#### Ammonium oxidation

The oxidation of NH_4_^+ ^was considered in the simulations to account for an increase (~0.02 mM) in nitrate concentrations observed at the onset of ureolysis. The oxidation was attributed to nitrification, which was approximated by the following overall reaction (assuming no NO_2_^- ^buildup):

(12) $${\text{NH}}_{4}^{+}+2{\text{O}}_{2\left(\text{aq}\right)}\to {\text{NO}}_{3}^{-}+{\text{H}}_{2}\text{O + }2{\text{H}}^{\text{ + }}$$

The overall rate was described using Monod kinetics:

(13) $$R_{NH_{4}^{+}}=\frac{\mu_{\max}}{Y_{bio}}C_{bio}\frac{C_{NH_{4}^{+}}}{K_{NH_{4}^{+}}+C_{NH_{4}^{+}}}\frac{C_{O_{2\left(aq\right)}}}{K_{O_{2\left(aq\right)}}+C_{O_{2\left(aq\right)}}}$$

Half-saturation constants *K_NH4+ _*(1.48 × 10^-5 ^mol/L) and *K_O2(aq) _*(2.41 × 10^-5 ^mol/L), and the rate μ_max _(9.53 × 10^-6 ^s^-1^) were taken from the literature [49]. The *C_bio_*/*Y_bio _*term was assumed constant and was calibrated (10^-15 ^mol/L). The dissolved oxygen concentration at the column inlet (CO_2_(aq) in Eq. 13) was set to 2 × 10-3 mol/L (~ 6 mg/L, close to atmospheric), in the range of measured concentrations. These concentrations dropped by about half in the column effluent. Note that the experiment was carried out specifically under oxic conditions to avoid potential complications arising from oxygen depletion by microbial reactions. Because oxygen-limited conditions were not observed, and amounts of produced nitrate were consistent with the amount of oxygen decrease, other microbially-driven oxygen-consuming reactions (e.g. organic matter oxidation) did not appear to be limiting and thus were not included into the model for simplicity.

#### Ion exchange

Ion exchange was implemented for Na^+^, K^+^, NH_4_^+^, Ca^2+^, Mg^2+^, and Sr^2+^, using a generic exchanger (i.e., no specific minerals are associated with exchange). Ion selectivity coefficients for INL sediments from Baker et al. [50] were used, except for K^+^, for which no data were reported by these authors, and for which the same selectivity as for Na^+ ^was assumed. The implemented exchange model followed the Gapon convention [51] with Ca^2+ ^as the reference cation. The sediment cation exchange capacity was calibrated, yielding a value (11.9 cmol/kg) in line with that measured by Baker et al. [50] (21 cmol/kg) and other data cited by these authors (0.9-45 cmol/kg) for INL site materials.

## Results

### Aqueous geochemistry and microbial analysis

**Figure 3:**
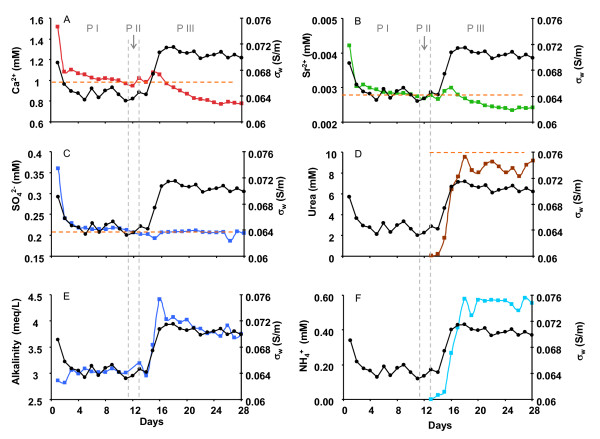
**Effluent concentrations of major ions of interest over time**. Black dot line represents fluid conductivity. The three phases (P) of the experiment are delineated by dashed vertical lines (P I: groundwater only; P II: molasses amendment; P III: urea amended groundwater). The orange dashed lines indicate influent concentrations when applicable.

Effluent geochemical data over the three phases of the experiment are shown in Figure 3.

Major ions monitored during phase I include Ca^2+ ^(Figure 3A); Sr^2+ ^(Figure 3B); and SO_4_^2- ^(Figure 3C). SO_4_^2- ^was monitored as an indicator of whether the system proceeded into sulfate reduction. During phase I, when site groundwater was pumped into the column, the major ions of concern (Ca^2+^, Sr^2+ ^and SO_4_^2-^) started at higher values compared to those measured later in this phase, when concentrations essentially matched the column inlet water. This was attributed to the effect of salt dissolution and ion desorption from the column matrix material. Alkalinity was relatively stable throughout this phase (Figure 3E). After ~4 days of equilibration with site groundwater, the column was stabilized with respect to most of the ions of interest including Ca^2+^, Sr^2+^, alkalinity and SO_4_^2- ^(Figure 3).

During phase II, groundwater containing 5 mg/L molasses was injected into the column to stimulate microbial activity. DO was monitored on a daily basis during molasses injection and was used as an indicator of microbial activity. The effluent DO was measured at 3.5, 2.2 and 1.7 mg/L during the first three days following molasses injection. Comparing to the constant influent value at ~6 mg/L, the decrease of DO indicated the increased microbial activity. Phase III commenced when the effluent DO level dropped to ~ 1 mg/L to prevent the system from entering denitrification and sulfate reduction conditions. No other major geochemical changes were observed during this phase.

For the final experimental Phase III, 10 mM urea amended groundwater (no molasses) was introduced into the column to initiate urea hydrolysis and calcium carbonate precipitation. Effluent geochemical monitoring revealed a decrease in urea concentration (compared to the influent level, Figure 3D), an increase of alkalinity (Figure 3E) and the production of NH_4_^+ ^(Figure 3F), all indicative of the occurrence of urea hydrolysis. However, the relatively high effluent urea concentration (average ~8.7 mM compared to influent at 10 mM, Figure 3D) indicated that only ~13% of the urea added was hydrolyzed. This hydrolysis rate was stable during phase III. A peak concentration was observed at ~ day 16 for Ca^2+ ^and Sr^2+ ^at the beginning of phase III, coinciding with the production of NH_4_^+^. This is likely due to ion exchange of the NH_4_^+ ^for sorbed Ca^2+ ^and Sr^3+ ^at mineral surfaces (Eq. 2). Following the short duration peaks, the concentrations of Ca^2+ ^and Sr^2+ ^decreased gradually to steady levels, with a 28% decrease for Ca^2+ ^(from 1.08 mM to 0.78 mM) and 20% decrease for Sr^2+ ^(from 3 μM to 2.4 μM) compared to influent concentrations during Phase III. The trend for alkalinity was similar to Ca^2+ ^and Sr^2+ ^with a short period of peak value upon the start of urea hydrolysis followed by a gradual decrease from 4.4 meq/L to 3.7 meq/L (a 16% decrease). The stoichiometric ratio between decreased alkalinity and Ca^2+ ^is ~ 2:1, consistent with precipitation of calcium carbonate according to Equation 3. Although an increase of pH is expected from urea hydrolysis according to equation 1, the pH of the effluent was stable at ~ 8.5 throughout the experiment (data not shown), partially due to pH buffering by the sediments. As mentioned previously, this pH value at 8.5 was higher than those observed in the VZRP where the groundwater used in this experiment was acquired, probably due to CO_2 _out gassing to the atmosphere. SO_4_^2- ^concentrations were stable throughout Phase III, indicating no occurrence of sulfate reduction (Figure 3C).

Fluid conductivity (*σ_w_*) is sensitive to changes in total dissolved solids (TDS) and a distinctive pattern of *σ_w _*change was observed during the different phases of the experiments that correlated well with geochemical data (Figure 3). The initial effluent *σ_w _*(~ 0.07 S/m) from the column was higher than that of the groundwater (0.062 S/m) due to the accumulation of dissolved and desorbed ions in the pore water before reaching steady state. *σ_w _*reached steady state in ~ 4 days and stayed relatively stable throughout the remainder of Phase I (Figure 3). During Phase II, the small concentration of molasses (5 mg/l) added had minimal impact on *σ_w_*. During phase III (urea injection) the fluid conductivity initially increased relatively rapidly from 0.064 S/cm to 0.0714 S/m (~12% increase), and then slowly decreased although it still remained elevated at the end of this phase.

The total volume and surface area of calcium carbonate precipitated was estimated. A total of 4.8 liter of urea amended solution was pumped through the large column during Phase III. Based on the Ca^2+ ^concentration decrease in the effluent, the amount of calcium carbonate precipitated was estimated at an average of 0.12 millimole per liter of solution, resulting in a total of 0.058 g CaCO_3 _precipitation during the experiment. This resulted in a total volume of 0.021 cm^3 ^based on a density of 2.7 g/cm^3 ^for calcium carbonate. Assuming a uniform distribution of the precipitates within the site sediments, this is equivalent to a negligible volume (~ 0.007%) of the total pore space and one would also infer a negligible contribution to the change of total surface area. Hydraulic conductivity measurements made at the end of the experiment showed no changes relative to initial conditions, supporting the interpretation of limited calcium carbonate precipitation.

Microbial cell densities in sediments (fine portion) and pore fluid samples were counted under a fluorescent microscope after staining with acridine orange and fixation. In a dilute clay suspension (~ 0.1 g/l), the cell density in the sediment is much lower (1.3E5) than that of the pore water (1.9E6). Cell adherence analysis showed that most of the cells that appeared attached to the sediments detached easily. This indicates either poor affinity of the microbial cells to sediment particles or the possibility that these cells were from the fluid but artificially attached/appeared attached to sediments during sample preparation/fixation.

### Electrical Geophysical response

**Figure 4:**
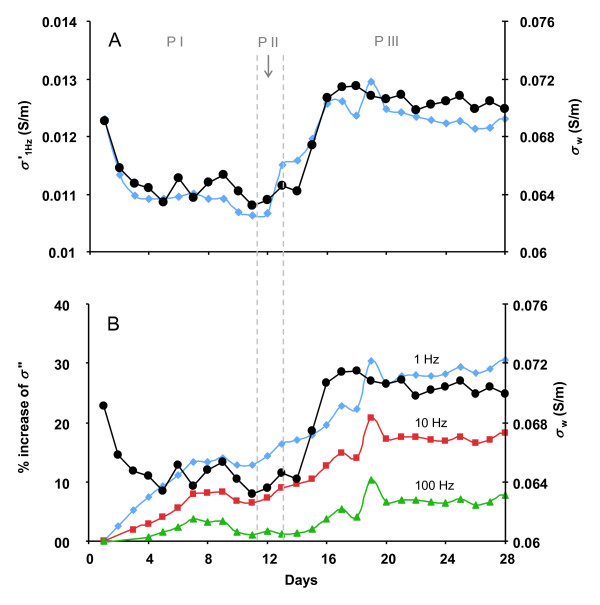
**Complex conductivity measurements (A) *σ*' at 1 Hz and (B) *σ*" at 1, 10 and 100 Hz during the experiment, both plotted together with the fluid conductivity data (black dots)**.

Complex conductivity measurements (both *σ' *and *σ"*) acquired during the experiment revealed electrical signal changes associated with the physicochemical transformations during enzymatically catalyzed urea hydrolysis (Figure 4).

Figure 4 shows the changes of *σ' *and *σ" *during the experiment calculated based on the phase shift (*Φ*) and magnitude (|*σ|*) measurements, as shown in equations 6 & 7. As previously discussed, *σ' *is controlled primarily by charge transfer through interconnected fluid filled pore spaces in natural sediments. Our data shows that the changes of *σ' *(at 1 Hz shown in Figure 4A) followed the changes of *σ_w _*throughout the experiment, indicating the dominant control of *σ_w _*on bulk conductivity. During Phase I, when groundwater was pumped into the column for initial equilibration, both *σ' *and *σ_w _*experienced sharp decreases before reaching a steady state. No significant changes were observed during molasses injection (Phase II). Both *σ' *and *σ_w _*increased concurrently following urea injection in Phase III. After the first few days in Phase III, however, both *σ' *and *σ_w _*began gradually decreasing during the course of urea injection, although the values remained elevated relative to the end of Phase I and to Phase II.

As discussed previously, *σ" *represents charge polarization associated with the EDL and is affected by the charge structure of the specific surfaces of the solid phases determined by mineralogy as well as pore fluid properties. In our experiment *σ" *increased step-wise throughout the experiment at all frequencies (Figure 4B). Percent changes of s" are plotted in Figure 4B for easier comparison between the three frequencies (1, 10 and 100 Hz). During Phase I, *σ" *increased steadily for ~ 7 days before it leveled out for the reminder of Phase I and II. Although changes of electrical signatures associated with the introduction of microbial cells have been observed [52], no clear evidence of microbial effect was observed during phase II when the microbial activity was stimulated with molasses. Additional increase of *σ" *occurred at the beginning of Phase III in parallel with the increase of *σ_w _*due to urea hydrolysis. Once urea hydrolysis and *σ_w _*reached steady states, *σ" *was stabilized and was maintained at a relatively constant level throughout the remainder of Phase III (Figure 4B). The actual values of the phase and imaginary conductivities before and after the experiments are listed in table 2. A comparison of *σ" *trend at three different frequencies also shows a more significant increase at low frequencies (30 ± 7%, 18 ± 4% and 7 ± 3% at 1, 10 and 100 Hz, respectively).

**Table 2:** Changes of the phase and imaginary conductivity during the experiment

Frequency (Hz)	*σ*" (S/m)		Percent change	φ (mRads)		Percent change
				
	Before	After		Before	After	
1	5.1E-05	6.7E-05	30.6	4.1	5.4	30.6
10	8.5E-05	1.0E-04	18.2	6.9	8.1	18.0
100	1.2E-04	1.3E-04	7.7	9.5	10.3	7.4

### Seismic results

**Figure 5:**
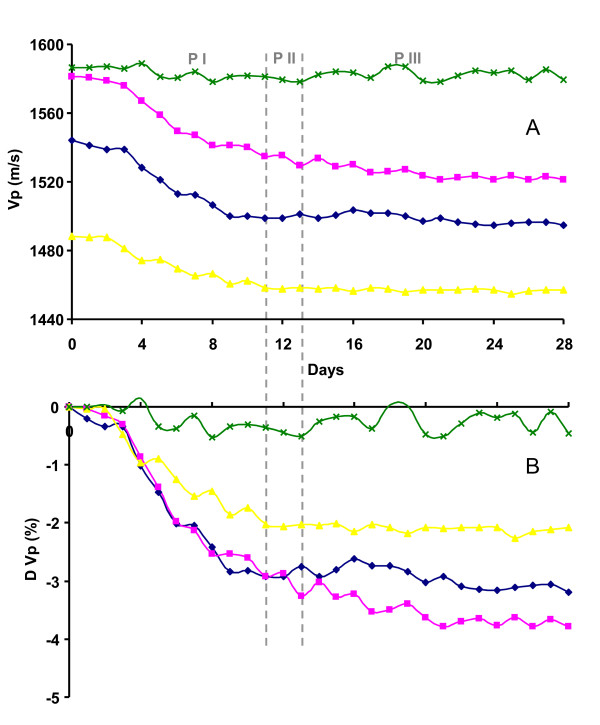
**P-wave seismic transmission measurements during the stimulation experiment**. Panel A shows the measured P-wave velocity at 4 depth levels in the column (Level 1: Blue; Level 2: Pink; Level 3: Yellow; Level 4: Green) as a function of elapsed time. Panel B shows percent change in velocity.

P-wave seismic transmission measurements acquired during the column experiment indicated only small changes in seismic properties and no direct signature of the stimulation of urea hydrolysis and calcium carbonate precipitation. Figure 5 shows the results of seismic measurements at the 4 discrete depth levels. Levels 1, 2, and 3 are measuring property changes in the native sediment while level 4 sampled the sand at the bottom of the column. Baseline velocities varied between 1600 and 1450 m/s with variations attributed to packing heterogeneity and gravel distribution. As can be seen in panel A of Figure 5, P-wave velocities displayed an initial gradual reduction for the transducer levels sampling the INL sediment during Phase I, while the sand showed an almost constant velocity of 1590 m/s. The maximum velocity reduction is ~ 60 m/s (~ 4%) at level 2 (Panel B). No signals correlated with either the molasses injection (Phase II) or the urea injection (Phase III).

### Synchrotron micro-CT Imaging Results

Synchrotron micro-CT imaging was conducted on the small column after the initial packing and then again after 2 weeks of urea injection. While a small amount of soil disturbance was noted at several points in the column, the granular pack was largely undisturbed. Positioning repeatability on the order of 20 microns was observed and manual registration was utilized to translate the volume for comparison.

**Figure 6:**
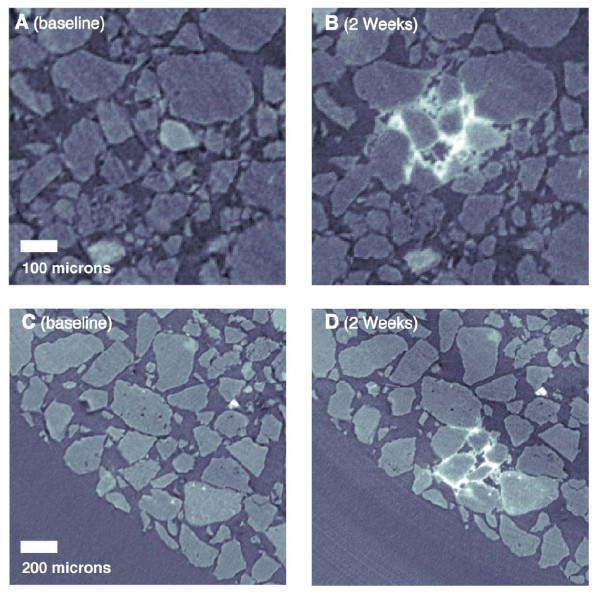
**Time-lapse synchrotron micro-CT images of precipitate formation**. Panels A and C show two sections of the micro column before urea treatment. Panels B and D show the same two sections after 2 weeks of urea injection. Localized regions of grain-coating precipitation are visible.

Figure 6 shows micro-CT imagery from 2 regions within the column before and after urea treatment. Several localized regions with high density precipitates are visible in the repeat scan; these patches are typically 200-500 microns in width, encompassing several grains. Within these regions, the precipitates manifested as grain coatings with no preference shown for grain-to-grain contacts. However, the number of precipitate patches is relatively small; the entire column contained only 15 zones with mineralization resolvable using micro-CT. No patches with similar characteristics were visible in the baseline images. There was no apparent preferential distribution of precipitation patches within the column overall, i.e. they occurred throughout the column rather than clustering near the inlet or column boundaries.

**Figure 7:**
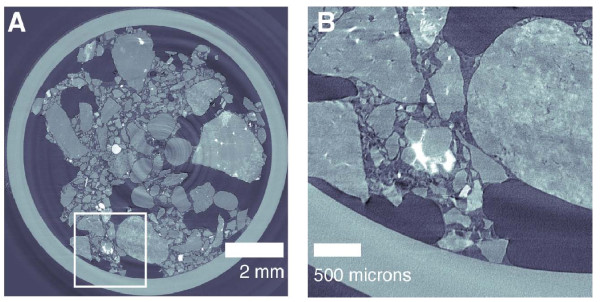
**Selected micro-CT slice of sub-core extracted from large column**. Panel A shows the cross-section of the entire sub-core while panel B shows a zoom of the small inset with a visible zone of localized mineralization.

Due to uncertainty as to whether the micro-column experiment was capturing the same precipitation processes as the larger column, a sub-core was extracted from the large column after completion of the urea treatment by insertion of rigid polycarbonate tubing. This sub-core was scanned at the same resolution as the micro-column (4.47 micron voxels) for direct comparison of precipitate architecture. Figure 7, panel A shows a CT cross-section of the entire sub-core; the large gaps in the sample are caused by the presence of small cobbles in the sediment which disrupted the fabric during coring, likely destroying some evidence of mineralization. However, careful examination of less disrupted zones within the core revealed high density features (Figure 7 B) similar to the observation of the small column (Figure 6 B, D).

An attempt was made to directly examine a precipitate patch by embedding the core in epoxy and thin-sectioning for micro-XRD analysis; unfortunately the patches identified via CT could not be recovered due to sediment fabric disruption during the embedding process.

### Reactive transport modeling results

**Figure 8:**
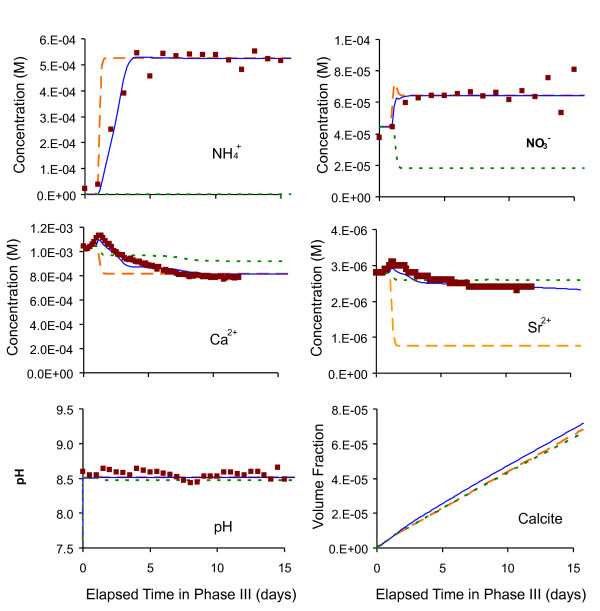
**Modeled concentration profiles (lines) and measured data (squares) in the column effluent, for cases considering the full model (solid lines), suppressing ion exchange (dashed lines), and suppressing ureolysis (dotted lines)**. Time zero represents the end of Phase II at ~13 days from the start of the experiment.

**Figure 9:**
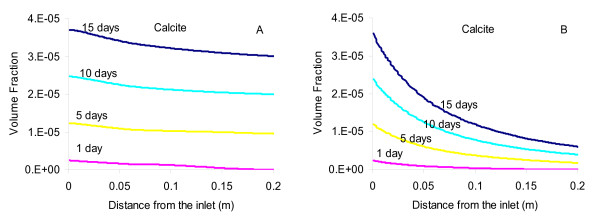
**Modeled calcium carbonate amounts within the column along its length (excluding the effluent) using (A) the full model and (B) without ureolysis**. Amounts computed for the case without ion exchange differ only slightly from results of the full model (see Figure 8).

Several reactive transport simulations were run to isolate effects of ion exchange and ureolysis. The model was run suppressing ion exchange in one case, and suppressing ureolysis in another case. The measured trends of key constituents were well captured by the full model (Figure 8), including sharp increases of NH_4_^+ ^from ureolysis (Eq. 1) and of NO_3_^- ^from NH_4_^+ ^oxidation (Eq. 12). Note that the sharp NH_4_^+ ^and NO_3_^- ^concentration fronts could only be modeled by iterating between transport and reaction computations. Early effects of ion exchange were reproduced reasonably well for Ca^2+ ^and Sr^2+^, showing small initial peaks in concentrations followed by a steady decline probably due to calcium carbonate precipitation reducing the TDS level in pore fluid (Figure 8). As would be expected, the case without ion exchange shows a sharper front for NH_4_^+^, no peaks in Ca^2+ ^and Sr^2+ ^concentrations, and much lower long term Sr^2+ ^concentrations in the effluent as less Sr^2+ ^is displaced by NH_4_^+^. The pH, which might have been expected to increase due to ureolysis (Eq. 1), was buffered by calcium carbonate precipitation (Eq. 3), as well as the imposed equilibration of the effluent with CO_2 _at near atmospheric conditions. The amount of calcium carbonate forming in the column was predicted to be quite small. Note that the original inlet solution (groundwater) was predicted to be supersaturated with respect to calcium carbonate by about half a saturation index unit. As a result, precipitation is predicted to occur throughout the entire column, in part from ureolysis within the column and also because of supersaturation prior to contact with the column sediments. The effect of ureolysis alone is apparent by comparing the results of simulations with and without ureolysis (Figure 9A and 9B), which show that 3 to 4 times more calcium carbonate is predicted to precipitate when ureolysis is active, compared to the case when it is inactive. Note that the computed calcium carbonate amounts in the effluent (Figure 8) after 15 days are nearly double those predicted within the column (Figure 9) because the imposed *p*CO_2 _at the column outlet (~10^-3.3 ^bar, to reflect near-atmospheric conditions) is lower than within the column (~10^-3 ^bar), resulting in additional calcium carbonate precipitation (e.g. 2HCO_3_^- ^+ Ca^2+ ^→ CaCO_3(s) _+ CO_2(g)_↑ + H_2_O).

## Discussion

The production of NH_4_^+ ^and loss of urea showed that we successfully stimulated urease activity in our experiment following the provision of molasses for a short period of time. Although no other nutrients were supplied during the injection of urea, effluent geochemical data showed sustained production of NH_4_^+^. However effluent geochemical data also indicated that only a fraction of the urea added was hydrolyzed (~13%). Calculations based on the observed changes in Ca^2+ ^concentrations, when excluding effects of ion exchange, and alkalinity indicated that only 0.12 millimole of calcium carbonate was precipitated per liter of solution used, which would have caused a negligible increase in the surface area of the sediments packed in the column. Additional contribution to calcium carbonate precipitation from Ca^2+ ^exchanged from the sediment is small even when the exchange rate was assumed at the peak level observed (~ Day 16) throughout the experiment. Unaltered hydraulic conductivity measured at the end of the experiment supported the small amount of calcium carbonate precipitation with minimal impacts on hydraulic properties.

The changes of *σ' *coincided linearly with the changes of *σ_w _*throughout the experiment (Figure 4A). This linear correlation between *σ*' and *σ_w _*agrees well with Archie's law [18] and indicated the dominant control of the pore fluid conductivity on the bulk electrical conductivity of the material. The most significant changes of *σ' *occurred at the beginning of the column experiment and the initiation of urea injection due to the significant changes in TDS associated with salt dissolution and/or ion desorption and urea hydrolysis, respectively. The slow decrease of both *σ*' and *σ_w _*during the latter portion of the urea injection period coincided with the slow decrease of Ca^2+^, Sr^2+ ^and alkalinity (Figure 3A, B and 3E), presumably due to the process of calcium carbonate precipitation.

The observed changes of *σ" *are more intriguing than the changes of *σ*'. As stated earlier, changes in the polarization (*σ"*) were expected during calcium carbonate precipitation because of the additional contribution to the total surface area from newly precipitated calcium carbonate [29]. However, an increase of *σ" *was not observed during the steady state of calcium carbonate precipitation in phase III, likely because the amount of calcium carbonate precipitation was small. This is in contrast to previous experiments where a much larger amount of calcium carbonate was produced and strong electrical responses were observed [29]. It is important to note however that in the previous experiments large glass beads were used rather than natural sediments; the latter have a large baseline surface area that provides a strong and dictating background electrical polarization signal and further reduces the sensitivity of electrical signals to small amounts of calcium carbonate precipitation.

Although not observed during the stable phase of calcium carbonate precipitation, we observed more significant changes in the polarization signal during the early stage of Phase I and III where significant changes of pore fluid chemistry occurred. Since *σ_w _*changed significantly during these periods, it is reasonable to consider the changes of pore fluid chemistry as the possible cause of the observed changes in *σ*".

With minimal changes to the sediment mineralogy, the observed changes in electrical phase response can be attributed to changes in EDL properties (e.g. charge density, charge mobility) in response to pore fluid chemical alterations. This has been explored by previous researchers investigating the electrical properties of porous media [20,21,24,53]. We focus our discussion on the initial stages of phase I and III because the most significant changes in both polarization and fluid chemistry occurred during these periods. Figure 3 shows clearly the fluid chemistry differences during these two phases: while the major ions during Phase I were typical of site groundwater, new species were produced in Phase III, primarily NH_4_^+^, due to urea hydrolysis. Although urea was also a new species introduced during Phase III, we assume minimal polarization effect from urea since it is presumably an uncharged species. During the early stage of Phase I, the concentrations of the major ions (for example Ca^2+ ^and SO_4_^2-^) decreased due to equilibration of the sediments with site groundwater. The initial high concentrations of the major ions were caused by salt dissolution and/or ion desorption from mineral surface as discussed above. Coincident with the decreases of the major ion concentrations was the continuous increase of the polarization signals (*σ"*). At the beginning of phase III, the major pore fluid chemical changes were the production of NH_4_^+ ^and increase of alkalinity. As discussed above, the production of NH_4_^+ ^enhanced ion exchange and caused the occurrence of the short peaks for Ca^2+ ^and Sr^2+^. A concurrent increase of *σ" *was observed during this period before reaching a steady state following the trend of change for NH_4_^+^. Comparison of the changes in both *σ" *and fluid conductivity between phase I and III reveals an inversed correlation: the increases of *σ" *in both phases, but the fluid conductivity was decreasing in phase I while increasing in phase III.

The reversed correlation between *σ" *and fluid conductivity between the two phases suggests inherent differences in the mechanisms of the polarization. The increase of *σ" *is an indication of the increased capacitive effects at the grain/fluid boundary. Previous studies have indicated a binary change of this capacitive effect for different ranges of ionic strengths; in particular, this capacitive effect was positively correlated with pore fluid ionic strength at low ionic strength, but negatively correlated at high ionic strengths [24]. The groundwater used in this experiments had an ionic strength of < 10 mM and generally falls into the low ionic strength range. Thus, the decrease of ionic strength (fluid conductivity) observed during the early stage of Phase I would have been predicted by Lesmes and Frye [24] to have resulted in a decrease of *σ"*, contradictory to our observations. Insights learned from P wave data could potentially explain this discrepancy. The P wave velocities decreased systematically at the sensor levels sampling the native sediment during the early stage of Phase I. If a high fraction of swelling clays, specifically smectite, was present, the P wave velocity decrease could have been due to a gradual hydration and expansion of clay particles trapped at grain contacts. The existence of significant smectite fractions at the INL site has been previously documented [41]. Such a hydration process would provide additional polarizable surface area that was previously not available and thus promote polarization behavior, causing increases in *σ"*. The fact that the zone of clean packing sand (level 4) did not exhibit the same velocity reduction suggests that a property change in the native sediment is responsible. The small but significant variation observed in baseline V_p _values at the different sensor depths was likely associated with heterogeneous packing of the sediments within the column as mentioned above.

The correlation between ionic strength (fluid conductivity) and *σ" *is positive during the early stage of Phase III, indicating a promoted polarization magnitude in this phase. We attribute this increase of *σ" *during this stage to (1) production of NH_4_^+ ^that could potentially modify sediment surface charge properties through cation exchange and (2) hydroxyl ion (produced during urea hydrolysis) adsorption/titration on the mineral surface due to the pH buffering capacity of the sediments.

As discussed previously, EDL polarization considers primarily the tangential movement of charges and the charge complexation structure of the EDL is expected to be relatively stable when pore fluid chemistry is unaltered. However, active ion exchange behavior occurred in this system during Phase III due to the production of NH_4_^+^, an ion that is readily exchangeable with surface complexed cations, including Na^+^, Ca^2+ ^and Sr^2+^. This ion exchange process could modify surface charge structure, including charge density and mobility and could potentially result in an increase of the polarization at the interface. The effects of ion exchange on electrical signature are less understood as has been observed and discussed by others [22,23]. In addition, hydroxyl ions produced during urea hydrolysis have a tendency for surface adsorption/titration due to the high pH buffering capacity of sediments with large fractions of fine particles. Hydroxyl adsorption/titration on sediment grains could enhance surface charge density and promote polarization behavior, as observed in other studies with porous media of high pH buffering capacity [54]. At the later stage of Phase III, ion exchange and hydroxyl adsorption/titration presumably reached a steady state. This stabilized the surface charge structure with NH_4_^+ ^acting as one of the new ionic species contributing to surface complexation. Manifested in *σ"*, the polarization magnitude was stabilized during this period.

The P-wave seismic measurements during phase III did not record an increase in velocity that would be indicative of grain cementation due to calcium carbonate precipitation. In light of the relatively small amount of CaCO_3 _precipitated and the localized nature of mineralization observed via micro-CT, this lack of a P-wave signature is not surprising. Previous observations of increases in frame shear stiffness during microbial urea hydrolysis [13] were associated with larger volume fractions of cement more evenly distributed across the pore space. The small changes in seismic response were likely driven by sample hydration and clay swelling rather than CaCO_3 _precipitation.

The dynamic micro-CT observations of the micro-column and the extracted sub-core clearly exposed evidence of mineralization. Since the precipitate observed was never directly interrogated using a method capable of resolving chemical composition, we have only indirect evidence that the mineralized patches were calcium carbonate; the combination of cation data and modeling suggests that this is likely the case. However, the occurrence of localized dissolution/re-precipitation reactions involving silicate minerals within the sediment matrix cannot be ruled out, but would be expected to proceed at a much slower rate and thus to a much more limited extent than calcium carbonate, and would not be expected to significantly impact effluent chemistry over the relatively short duration of the test. The pore scale geometry of precipitates appears to be largely grain coating, localized in small patches rather than homogeneously distributed. This distribution, in addition to the minimal porosity reduction, likely contributes to the lack of observed changes in seismic P-wave velocity and hydraulic conductivity following urea hydrolysis. A large seismic signature would indicate a significant increase in mean contact stiffness, which would require precipitate formation at a larger number of grain contacts.

After taking into account ion exchange and atmospheric equilibration processes, the latter of which induced premature calcium carbonate precipitation and ammonia oxidation, the reactive transport model successfully simulated the concentration changes of major constituents during urea hydrolysis and calcium carbonate precipitation. The ion exchange processes associated with NH_4_^+ ^production were evidenced by effluent geochemical data and successfully captured in the model. Changes in TDS calculated from variations of the concentrations (measured and predicted) of major ionic species in the pore fluid correlated well with observed bulk electrical conductivity measurements; the change of both parameters was ~10% - 12% during steady state of Phase III. Although the extent of urea hydrolysis was low (only ~13% of the added urea was hydrolyzed), compared to predicted baseline levels the calcium carbonate precipitation was enhanced 3 to 4 times by the urea addition, confirming the successful promotion of calcium carbonate precipitation processes during the experiment. Research has indicated that attached microbial cells are responsible for a much greater fraction of urea hydrolysis than planktonic cells [8]. The low hydrolysis rate in our column was probably associated with a low density of attached cells in the sediments based on cell count results presented above. As was reported earlier, the sediments used in the experiment were samples that had been stored dry in archive for over 10 years. Diminished microbial activity would have been expected. Although fresh groundwater was used to rehydrate the sediments, the attachment of cells to the sediments appeared to be limited based on cell adherence tests. Dissolution of Ag and AgCl from the electrodes used for geophysical measurements might have further inhibited urease activity [55] and have been observed by other researchers with extracellular urease (personal communication, George Redden, INL).

## Conclusion

In this study, multiple geophysical methods including electrical, seismic and micro-CT, together with reactive transport modeling, were evaluated for their utility in monitoring and characterizing ureolysis promoted calcium carbonate precipitation for trace metal sequestration. Urease activity was successfully stimulated in columns of natural sediment and groundwater following the introduction of molasses as a nutrient source. The rate of urea hydrolysis was low, probably due to the low density of microbes in the sediments and perhaps the toxicity of Ag from electrodes. However, this low ureolytic activity still led to a calcium carbonate precipitation rate that was 3-4 times higher than the predicted baseline, supporting the potential of remediation approaches based on ureolytically-driven calcium carbonate precipitation for sequestration of divalent trace metal and radionuclides. Although seismic monitoring was not able to detect changes during the calcium carbonate precipitation phase of the experiment due to minimal changes to the bulk modulus, complex conductivity measurements successfully detected changes in pore fluid chemistry during urea hydrolysis. Electrical conductivity correlated well with TDS changes due to urea hydrolysis and was successfully captured by effluent geochemical data and simulated by reactive transport modeling. Important for field monitoring of urea hydrolysis, we believe that the imaginary component of the electrical geophysical signal was related to the alteration of surface charge structure due to ion exchange from the production of NH_4_^+ ^and possibly hydroxyl adsorption to sediment surfaces or titration of surface proton sites. Previous observations of complex conductivity signatures directly from calcium carbonate precipitation [29] were not observed in this experiment presumably due to the small quantity of precipitated calcium carbonate, thus the small increase in surface area, relative to the volume of background sediments. Micro-CT imaging demonstrated the sparseness of calcium carbonate precipitation in the column and revealed the patchy and pore filling natures of the mineral precipitates. Micro-CT was shown to be a useful tool for dynamic imaging of the column to acquire direct evidence of precipitation geometry and distribution within the porous media. One dimensional reactive transport modeling with TOUGHREACT successfully simulated changes of major ion concentrations during the experiment, and the calculated TDS changes of pore fluid based on the model and effluent geochemical data correlated well with electrical conductivity measurements. The reactive transport modeling validated the reaction network outlined in Eq. 1-4 and demonstrated the usefulness of this model for simulating urea hydrolysis and calcium carbonate precipitation under field relevant conditions.

Our experiments uniquely combined complex electrical, seismic, and micro-CT imaging technologies with reactive transport modeling to study urea hydrolysis coupled with calcium carbonate precipitation under field relevant conditions. Indirect geophysical measurements provided a quick indicator of system transitions between different phases while geochemical and modeling results offered detailed information on critical geochemical processes and parameters, such as ion exchange and changes in TDS. In addition to elucidating the evolution of the pore fluid geochemistry, the modeling was particularly helpful in understanding intriguing geophysical signatures (e.g. imaginary conductivity) that were otherwise difficult to interpret. In addition, the precipitation end products inferred from all of these datasets were validated through micro-CT imaging that provided direct evidences of physical changes, including precipitate distribution and geometry. Although the limited extent of urea hydrolysis and calcium carbonate precipitation limited the demonstration of the complementary nature of these tools, our study showed clearly the advantages of combining multiple approaches to understand complex biogeochemical processes in the subsurface.

## Competing interests

The authors declare that they have no competing interests.

## Authors' contributions

YW, JBA-F and KHW designed and coordinated experimental execution and geophysical and geochemical sampling and analysis. NS and GZ carried out reactive transport modeling. YW, JBA-F and NS drafted the manuscript. KHW and JT provided aqueous analysis and assisted with data interpretation. SSH, YF and RS provided assistance in drafting and finalizing the manuscript. All authors read and approved the final manuscript and contributed to the revision.

## References

[B1] DOE Strength through Science: Powering the 21st Century 2000

[B2] Ferris GF Phoenix V Fujita Y Smith RW Kinetics of calcite precipitation induced by ureolytic bacteria at 10 to 20°C in artificial groundwater Geochim Cosmochim Acta 2004 67 8 1701 1722

[B3] Curti E Coprecipitation of radionuclides with calcite: Estimation of partition coefficients based on a review of laboratory investigations and geochemical data Appl Geochem 1999 14 433 445 10.1016/S0883-2927(98)00065-1

[B4] Zachara JM Cowan CE Resch CT Sorption of divalent metals on calcite Geochim Cosmochim Acta 1991 55 1549 1562 10.1016/0016-7037(91)90127-Q

[B5] Pingitore NE Eastman MP The coprecipitation of Sr^2+ ^with calcite at 25 degree C and 1 atm Geochim Cosmochim Acta 1986 50 10 2195 2203 10.1016/0016-7037(86)90074-8

[B6] Fujita Y Redden GD Ingram JC Cortez MM Ferris GF Smith RW Strontium incorporation into calcite generated by bacterial ureolysis Geochim Cosmochim Acta 2004 68 15 3261 3270 10.1016/j.gca.2003.12.018

[B7] Mitchell AC Ferris GF The coprecipitation of Sr into calcite precipitates induced by bacterial ureolysis in artificial groundwater: Temperature and kinetic dependence Geochim Cosmochim Acta 2005 69 17 4199 4210 10.1016/j.gca.2005.03.014

[B8] Fujita Y Taylor JL Wendt LM Reed DW Smith RW Evaluating the Potential of Native Ureolytic Microbes To Remediate a ^90^Sr Contaminated Environment Environ Sci Technol 2010 44 7652 7658 10.1021/es101752p 20815389

[B9] Wood WW Low WH Aqueous geochemistry and diagenesis in the Eastern Snake River Plain aquifer system Geol Soc Am bull 1986 97 1456 1466 10.1130/0016-7606(1986)97<1456:AGADIT>2.0.CO;2

[B10] Whiffin VS van Paassen LA Harkes MP Microbial Carbonate precipitation as a soil improvement technique Geomicrobiol J 2007 24 5 417 423 10.1080/01490450701436505

[B11] Druckenmiller ML Marote-Valer MM Hill M Investigation of Carbon Sequestration via Induced Calcite Formation in Natural Gas Well Brine Energy & Fuels 2006 20 172 179 10.1021/ef050115u21941670

[B12] Fujita Y Ferris GF Lawson DR Coldwell FS Smith RW Calcium Carbonate Precipitation by Ureolytic Subsurfface Bacteria Geomicrobiol J 2000 17 2

[B13] DeJong JT Fritzges MB Nusslein K Microbially induced cementation to control sand response to underained shear J Geotech Geoenviron Eng 2006 132 11 1381 1392 10.1061/(ASCE)1090-0241(2006)132:11(1381)

[B14] Warren LA Maurice PA Parmar N Ferris GF Microbially Mediated Calcium Carbonate Precipitation: Implications for Interpreting Calcite Precipitation and for Solid-Phase Capture of Inorganic Contaminants Geomicrobiol J 2001 18 1 93 115 10.1080/01490450151079833

[B15] Fujita Y Taylor JL Gresham TL Delwiche ME Coldwell FS McLing TL Petzke LM Smith RW Stimulation Of Microbial Urea Hydrolysis In Groundwater To Enhance Calcite Precipitation Environ Sci Technol 2008 42 8 3025 3032 10.1021/es702643g 18497161

[B16] Williams KH Kemna A Wilkins MJ Druhan J Arntzen E N'Guessan L Long P Hubbard SS Banfield J Geophysical monitoring of coupled microbial and geochemical processes during stimulated subsurface bioremediation Environ Sci Technol 2009 10.1021/es900855j 19764240

[B17] Hubbard SS Williams KH Conrad ME Faybishenko B Peterson J Chen J Long P Hazen T Geophysical monitoring of hydrological and biogeochemical transformations associated with Cr(VI) bioremediation Environ Sci Technol 2008 42 3757 3765 10.1021/es071702s 18546719

[B18] Archie GE The electrical resistivity log as an aid in determining some reservoir characteristics: Transactions of the American Institute of Mineral Metallurgy and Petroleum Engineers 1942 146 54 62

[B19] Wong J An electrochemical model of the induced-polarization phenomenon in disseminated sulfide ores Geophysics 1979 44 1245 1265 10.1190/1.1441005

[B20] Slater LD Choi J Wu Y Electrical properties of iron-sand columns: Implications for induced polarization investigation and performance monitoring of iron-wall barriers Geophysics 2005 70 4 G87 G94 10.1190/1.1990218

[B21] Schwarz G A theory of the low-frequency dielectric dispersion of colloidal particles in electrolyte solution J Phys Chem 1962 66 2636-2642

[B22] Olhoeft GR Low-frequency eletrical properties Geophysics 1985 50 12 2492 2503 10.1190/1.1441880

[B23] Vaudelet P Revil A Schmutz M Francesschi M Begassat P Induced polarization signatures of cations exhibiting differential sorption behaviors in saturated sands Water Resour Res 2011 47 W02526 21

[B24] Lesmes DP Frye KM Influence of pore fluid chemistry on the complex conductivity and induced polarization responses of Berea sandstone J Geophys Res(B Solid Earth) 2001 106 B3 4079 4090 10.1029/2000JB900392

[B25] Williams KH Ntarlagiannis D Slater LD Dohnalkova A Hubbard SS banfield JF Geophysical imaging of stimulated microbial biomineralization Environ Sci Technol 2005 39 19 7592 7600 10.1021/es0504035 16245832

[B26] Atekwana EA D. Dale Werkema J Duris JW Rossbach S Atekwana EA Sauck WA Cassidy DP Means J Legal FD In-situ apparent conductivity measurements and microbial population distribution at a hydrocarbon-contaminated site Geophysics 2004 69 1 56 63 10.1190/1.1649375

[B27] Ntarlagiannis D Williams KH Slater L Hubbard SS Low-frequency electrical response to microbial induced sulfide precipitation J Geophys Res (G Biogeosci) 2005 110 G02009

[B28] Lane JWJ Day-Lewis FD Casey CC Geophysical monitoring of a field-scale biostimulation pilot project Ground water 2006 44 3 430 443 10.1111/j.1745-6584.2005.00134.x 16681523

[B29] Wu Y Hubbard SS Williams KH Ajo-Franklin JB On the complex conductivity signatures of calcite precipitation J Geophys Res 2010 115 G00G04

[B30] Slater L Ntarlagiannis D Personna YR Hubbard S Pore-scale spectral induced polarization (SIP) signatures associated with FeS biomineral transformations Geophys Res Lett 2007 34 L21404

[B31] Personna YR Ntarlagiannis D Slater L Yee N O'Brien M Hubbard S Spectral induced polarization and electrodic potential monitoring of microbially-mediated iron sulfide transformations J Geophys Res (G Biogeosci) 2008 113 G02020

[B32] Davis CA Pyrak-Nolte LJ Atekwana EA Werkema DD Haugen ME Microbial-induced heterogeneity in the acoustic properties of porous media Geophys Res Lett 2009 36 L21405

[B33] Dvorkin J Berryman J Nur A Elastic moduli of cemented sphere packs Mech Mater 1999 31 461 499 10.1016/S0167-6636(99)00009-5

[B34] Pride S Berryman J Linear dynamics of double-porosity dual-permeability materials. I. Governing equations and acoustic attenuation Phys Rev E 2003 68 3 10.1103/PhysRevE.68.036603 14524908

[B35] Xu T Spycher NF Sonnenthal E Zhang GLZ Pruess K TOUGHREACT Version 2.0: A simulator for subsurface reactive transport under non-isothermal multiphase flow conditions Computers and Geosciences 2011 37 6 763 774 10.1016/j.cageo.2010.10.007

[B36] Spanne P Thovert JF Jacquin CJ Lindquist WB Johes KW Adler Pm Synchrotron Computed Microtomography of Porous Media: Topology and Transports Phys Rev Lett 1994 73 2001 2004 10.1103/PhysRevLett.73.2001 10056943

[B37] Stock S Microcomputed Tomography: Methodology and Application 2009 CRS Press: Boca Raton, FL

[B38] Noiriel C Bernard D Gouze P Thibault X Hydraulic properties and microgeometry evolution in the course of limestone dissolution by acidic water Oil Gas Sci Technol 2005 60 1-2 177 192

[B39] Englert A Hubbard SS Williams KH Li L Steefel CI Feedbacks between hydrological heterogeneity and bioremediation induced biogeochemical transformations Environ Sci Technol 2009 43 5197-5204 10.1021/es803367n 19708341

[B40] Winfield KA Spatial Variability of Sedimentary Interbed Properties Near the Idaho Nuclear Technology and Engineering Center at the Idaho National Engineering and Environmental Laboratory, Idaho 2003 Idaho Falls, Idaho

[B41] Bartholomay RC Mineralogical correlation of surficial sediment from area drainages with selected sedimentary interbeds at the Idaho Inational Engineering Laboratory, Idaho 1990 Water-Resources Investigations Report 90-4147; Idaho Falls, ID

[B42] Oliveira IB Demond AH Salehzadeh A Packing of sands for the production of homogeneous porous media Soil Sci Soc Am J 1996 60 1 49 53 10.2136/sssaj1996.03615995006000010010x

[B43] Lunau M Kemke A Walther K Martens-Habbena W Simon M Determining adherence of cells to sediment particles Environ Microbiol 2005 10.1111/j.1462-2920.2005.00767.x 15946292

[B44] Palandri J Kharaka YK A compilation of rate parameters of water-mineral interaction kinetics for application to geochemical modeling 2004 US Geol. Surv 64

[B45] Doherty J PEST: Model-Independent Parameter Estimation 2008 Watermark Numerical Computing: Brisbane, Australia

[B46] Fidaleo M Lavecchia R Kinetic study of enzymatic urea hydrolysis in the pH range 4-9 Chem Bioche Eng Q 2003 4 311 318

[B47] Barkouki TH Martinez BC Mortensen BM Weathers TS DeJong JT Ginn TR Spycher NF Smith RW Fujita Y Forward and Inverse Bio-Geochemical Modeling of Microbially Induced Calcite Precipitation in Half-Meter Column Experiments Transport in Porous Media 2011

[B48] Lasaga AC Kinetic Theory in the Earth Sciences 1998 Princeton University Press 811

[B49] Maggi F Gu C Riley WJ Hornberger GM Venterea RT Xu T Spycher NF Steefel CI Miller NL Oldenburg CM A mechanistic treatment of the dominant soil nitrogen cycling processes: Model development, testing, and application J Geophys Res (G Biogeosci) 2008 113 G02016

[B50] Baker LL Strawn DG Smith RW Cation Exchange on Vadose Zone Research Park Subsurface Sediment, Idaho National Laboratory Vadose Zone J 2010 9 1 10 10.2136/vzj2009.0149

[B51] Sposito G The Gapon and the Vanselow selectivity coefficients Soil Sci Soc Am J 1977 41 1205 1206 10.2136/sssaj1977.03615995004100060040x

[B52] Ntarlagiannis D Yee N Slater L On the low-frequency electrical polarization of bacterial cells in sands Geophys Res Lett 2005 32 L24402

[B53] Wong J An electrochemical model of the induced-polarization phenomenon in disseminated sulfide ores Geophysics 1979 44 7 1245 1265 10.1190/1.1441005

[B54] Zhang C Johnson TC Slater L Redden GD Spectral induced polarization signatures of hydroxyl adsorption in porous media 2010 American Geophysical Union, San Francisco, Ca Abstract # NS31B-1398

[B55] Chaperon S Sauve S Toxicity interaction of metals (Ag, Cu, Hg, Zn) to urease and dehydrogenase activities in soils Soil Biol Biochem 2007 39 2329 2338 10.1016/j.soilbio.2007.04.004

